# Sacubitril/Valsartan for heart failure: A protocol for systematic review and meta-analysis

**DOI:** 10.1097/MD.0000000000029149

**Published:** 2022-06-10

**Authors:** Wenqin Dai, Jinlan Luo, Xianli Huang

**Affiliations:** Department of Cardiology, Wuhan Wuchang Hospital, Wuchang Hospital Affiliated to Wuhan University of Science and Technology, Hubei, China.

**Keywords:** heart failure, meta-analysis, mortality, sacubitril–valsartan, systematic review

## Abstract

**Background:**

Sacubitril–valsartan has been shown to have superior effects over angiotensin-converting enzyme inhibitors and angiotensin receptor blockers in patients with heart failure (HF). However, the effects of sacubitril–valsartan have never been systematically evaluated. Therefore, we performed a protocol for systematic review and meta-analysis to evaluate the efficacy and safety of sacubitril–valsartan in patients with HF.

**Methods:**

We selected 8 databases, including PubMed, the Web of Science, Embase, Cochrane Library, the Chinese National Knowledge Infrastructure, the Chinese Science Journal Database, Wanfang Data, and the Chinese Biomedical Literature Database. The search time was from database establishment to March 2022. Two reviewers will screen the records and include quality studies according to inclusion criteria independently. Two reviewers will assess the risk of bias of the included studies by the “Risk of Bias Assessment Tool” of the Cochrane Handbook for randomized controlled trials. Statistical analysis will be performed with Review Manager software 5.3.

**Results:**

A synthesis of current evidence of sacubitril–valsartan for treating HF will be provided in this protocol.

**Conclusion:**

The results of this study will provide a theoretical basis for the clinical use of sacubitril–valsartan to treat HF.

## 1 Introduction

Heart failure (HF) is a syndrome characterized by symptoms (such as breathlessness, ankle swelling, and fatigue) and signs (e.g., raised jugular venous pressure, pulmonary crackles, and peripheral edema) caused by structural or functional cardiac abnormalities that lead to elevated intracardiac pressures or a reduced cardiac output at rest or during stress.^[1–3]^ It is a leading and increasing cause of morbidity and mortality worldwide. The prevalence of HF is age-dependent, ranging from <2% of people younger than 60 years to >10% of those older than 75 years.^[4]^ As a result of ageing of the general population and improved treatment of acute cardiovascular events, the prevalence of heart failure is projected to increase by 25% in the next 20 years.^[4]^ Several drugs have been applied to HF, such as β blockers, calcium-channel blockers, angiotensin-converting enzyme (ACE) inhibitors, and angiotensin receptor blockers (ARBs), but there was no obvious efficacy.^[6–9]^

Sacubitril–valsartan is a first-in-class angiotensin receptor neprilysin inhibitor that has been used in both HF and hypertension.^[10,11]^ This neprilysin inhibitor has vasodilating effects and facilitates sodium excretion, and when combined with the inhibition of the renin-angiotensin system, it has superior effects over ACE inhibitors or ARBs alone. Published studies have indicated that sacubitril–valsartan significantly reduced the pooled endpoints of all-cause mortality, cardiovascular death, and hospitalization for HF compared with enalapril for HF.^[12]^ However, several studies have shown that sacubitril–valsartan did not result in significantly lower rates of rehospitalization for HF, death from cardiovascular causes, and death from all causes, especially in HF with preserved ejection fraction.^[13]^ Although the risk of serious angioedema from neprilysin inhibition has been minimized, major adverse events, including hypotension, worsening renal function, and hyperkalemia, have been shown to be heterogeneous in different randomized controlled trials (RCTs). Therefore, we performed a protocol for systematic review and meta-analysis to evaluate the efficacy and safety of sacubitril–valsartan in patients with HF.

## 2 Methods

### 2.1 Study registration

This systematic review has been registered on Open Science Framework (Registration number: DOI 10.17605/OSF.IO/CD69V) and will be performed according to the Preferred Reporting Items for Systematic Reviews and Meta-Analysis Protocol (PRISMA-P) statement guidelines.^[14]^ Ethical approval was not required for this study as all the research materials are derived from published studies.

### 2.2 Selection criteria

The efficacy and safety outcomes of sacubitril–valsartan were compared with those of ACE inhibitors or ARBs in all RCTs. The following inclusion criteria were used: RCTs with a sacubitril–valsartan group and a control group; RCTs including chronic or hemodynamically stable patients with acute HF; and RCTs analyzing primary efficacy outcomes, including death from cardiovascular causes, death from any cause, hospitalization for HF, and key adverse events, including symptomatic hypotension, worsening renal function, hyperkalemia, and angioedema. The exclusion criteria were as follows: duplicated papers related to the same trial; studies, such as systemic reviews, comments, case reports, conference abstracts, editorials, and observational cohort studies; and incomplete RCTs or RCTs failing to report the outcomes of interest.

### 2.3 Search strategy

We selected 8 databases, including PubMed, the Web of Science, Embase, Cochrane Library, the Chinese National Knowledge Infrastructure, the Chinese Science Journal Database, Wanfang Data, and the Chinese Biomedical Literature Database. The search time was from database establishment to March 2022. Search only Chinese and English literature. The database search was carried out in the form of subject headings combined with free words. The search terms included “Sacubitril/Valsartan,” “heart failure,” and “randomized controlled trial.” In addition, references to the included literature were traced back to supplement the acquisition of relevant literature.

### 2.4 Selection of studies

Two reviewers will independently review the titles, abstracts, and full text of the studies for eligibility for inclusion in this systematic review. All studies searched by electronic databases and identified by hand will be organized in Endnote X7 (Thomson Reuters, New York, NY). Any disagreement between the 2 reviewers will be resolved by discussion and consensus. If necessary, a third reviewer will intervene and resolve any disagreement.

### 2.5 Data extraction

Two review authors will independently extract the data and fill out the standard data extraction form, which includes study information such as the first author, publication year, title, journal name, research design, number of patients, inclusion criteria, interventions, control, treatment period, outcome measures, and adverse events. Data extraction will be performed by 2 independent investigators according to a predesigned review form. Disagreements are resolved through discussion among all authors.

### 2.6 Risk of bias assessment

Two reviewers will assess the risk of bias of the included studies by the “Risk of Bias Assessment Tool” of the Cochrane Handbook for RCTs.^[15]^ The evaluation contents include random sequence generation, allocation concealment, blinding of participants and personnel, blinding of outcome assessment, incomplete outcome data, selective reporting, and other biases. Each item is divided into “high risk,” “unclear risk,” and “low risk.” Any inconsistencies will be determined in consultation with the third reviewer.

### 2.7 Data synthesis and analysis

Statistical analysis will be performed with Review Manager software 5.3. Relative risk is used to evaluate the effect size for binary variables, and the mean difference is used as the efficacy analysis statistic for continuous variables. Heterogeneity between results will be assessed by the value of *P* and *I*^2^. When *P* > .1, *I*^2^ < 50%, it will be considered as no significant heterogeneity between the trials, and the fixed-effect model will be applied for statistics, otherwise, the random-effect model will be chosen.

### 2.8 Sensitivity analysis

If the risk of bias of the studies is high, sensitivity analysis will be performed to investigate the asymmetry of funnel plots to exclude low-quality studies.

### 2.9 Reporting bias

If the number of included RCTs is ≥9, an inverted funnel plot will be drawn to judge the publication bias of the included studies.

## 3 Discussion

The main pathogenesis of HF is related to renin angiotensin aldosterone system (RAAS), sympathetic nervous system, and natriuretic peptide system. In the early stage of the disease, the activation of RAAS and sympathetic nervous system can play a compensatory role in the heart.^[16]^ However, if they are activated continuously for a long time, they will promote the necrosis of myocardial cells, induce ventricular remodeling, and further progress and deterioration of cardiac function until death.^[17]^ Sacubitril–valsartan can inhibit the activation of RAAS system, enkephalinase, and the degradation of natriuretic peptide. It should augment this endogenous defence mechanism and could be beneficial in HF with both reduced and preserved ejection fraction. There is, however, insufficient evidence to prove any significant efficacy of sacubitril–valsartan compared to ACE inhibitors or ARBs alone. This systematic review and meta-analysis will provide a convincing conclusion to justify the efficacy and safety of sacubitril–valsartan in patients with HF. The conclusion of this review is anticipated to assist clinicians regarding the treatments of HF and benefits corresponding patients. These clues and conclusion are hoped to encourage researchers to conduct further research on the subject.

## Author contributions

**Conceptualization:** Wenqin Dai, Jinlan Luo.

**Data curation:** Jinlan Luo.

**Formal analysis:** Jinlan Luo.

**Funding acquisition:** Xianli Huang.

**Investigation:** Jinlan Luo.

**Methodology:** Xianli Huang.

**Project administration:** Xianli Huang.

**Software:** Xianli Huang.

**Writing – original draft:** Wenqin Dai.

**Writing – review & editing:** Xianli Huang.
